# The Social Distancing Imposed To Contain COVID-19 Can Affect Our Microbiome: a Double-Edged Sword in Human Health

**DOI:** 10.1128/mSphere.00716-20

**Published:** 2020-09-16

**Authors:** Célia P. F. Domingues, João S. Rebelo, Francisco Dionisio, Ana Botelho, Teresa Nogueira

**Affiliations:** a cE3c—Centre for Ecology, Evolution and Environmental Changes, Faculdade de Ciências da Universidade de Lisboa, Lisbon, Portugal; b INIAV - National Institute for Agrarian and Veterinary Research, Bacteriology and Mycology Laboratory, Oeiras, Portugal; U.S. Department of Energy Joint Genome Institute

**Keywords:** microbiome, COVID-19, social distancing, person-to-person transmission, dysbiosis, bacterial diversity, antibiotic resistance

## Abstract

Hygienic measures imposed to control the spread of severe acute respiratory syndrome coronavirus 2 (SARS-CoV-2) and contain COVID-19 have proven effective in controlling the pandemic. In this article, we argue that these measures could impact the human microbiome in two different and disparate ways, acting as a double-edged sword in human health. New lines of research have shown that the diversity of human intestinal and oropharyngeal microbiomes can shape pulmonary viral infection progression. Here, we suggest that the disruption in microbial sharing, as it is associated with dysbiosis (loss of bacterial diversity associated with an imbalance of the microbiota with deleterious consequences for the host), may worsen the prognosis of COVID-19 disease. In addition, social detachment can also decrease the rate of transmission of antibiotic-resistant bacteria. Therefore, it seems crucial to perform new studies combining the pandemic control of COVID-19 with the diversity of the human microbiome.

## Abstract (precis)

Hygienic measures imposed to control the spread of severe acute respiratory syndrome coronavirus 2 (SARS-CoV-2) and contain COVID-19 have proven effective in controlling the pandemic. In this article, we argue that these measures could impact the human microbiome in two different and disparate ways, acting as a double-edged sword in human health. New lines of research have shown that the diversity of human intestinal and oropharyngeal microbiomes can shape pulmonary viral infection progression. Here, we suggest that the disruption in microbial sharing, as it is associated with dysbiosis (loss of bacterial diversity associated with an imbalance of the microbiota with deleterious consequences for the host), may worsen the prognosis of COVID-19 disease.

## PERSPECTIVE

Among the main recommendations to fight the COVID-19 pandemic are to avoid interpersonal contacts, to disinfect hands upon touching physical surfaces in anthropogenic settings, and to follow strict respiratory etiquette rules to prevent the spread of viral particles and aerosols contaminated with severe acute respiratory syndrome coronavirus 2 (SARS-CoV-2). Quarantine and confinement are also indicated for the prevention of disease transmission in the case of individuals with suspected or confirmed infection.

Social interconnections between people, however, also lead to the sharing of those microorganisms that have been coevolving with humans and which are very important for human health maintenance, contributing to the control of many diseases and syndromes (1). The human microbiota engages in symbiotic or mutualistic relationships within the human body. The collection of its genomes—the microbiome—is estimated to account up to 99% of the unique genes in the human body (2) and provides genetic information to perform many complementary functions that are lacking in the human genome, such as helping to break down nutrients and molecules or to stimulate the immune system (3). Changes in the balance of the gut microbiome (dysbiosis) are associated with a greater susceptibility to diseases and opportunistic infections, due to the decrease in the protective microbial load of symbiotic bacteria, which can lead to dysregulation of the immune system and to autoimmune diseases (4). Such changes were also found to be correlated with COVID-19 prognosis (5, 6). Yet there are no data on the effect of human behavior during the COVID-19 pandemic on the gut microbiota.

## SOCIAL NETWORKING AND THE HUMAN MICROBIOME

**FIG 1:**
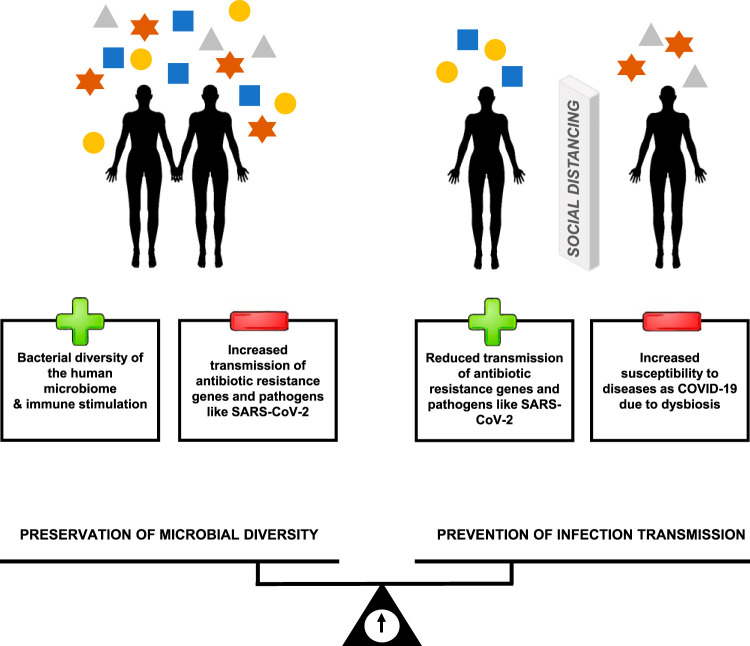
Schematic representation of the sharing of microorganisms of the human microbiome. On the left side, individuals are connected and share their microorganisms (the colored symbols represent microbial diversity), preserving the microbial diversity of their microbiomes but also the sharing of pathogens, such as COVID-19 and antibiotic-resistant bacteria. The right side represents social distancing, which is an important tool in preventing the spread of antibiotic-resistant bacteria and pathogens like SARS-CoV-2, but it can trigger dysbiosis leading to the appearance of opportunistic infections and a worse prognosis for COVID-19. The scale at the bottom represents the balance that should be reached between the preservation of the microbial diversity of the human microbiome and infection transmission prevention (both situations having pros and cons).

The sharing of microorganisms between humans helps to build up the human microbiome (Fig. 1). Microorganisms belonging to the oral, intestinal, and nasopharyngeal microbiomes can be transferred from person to person in a physical social network. People living in the same home, i.e., sharing a household, are more likely to harbor similar profiles of bacterial lineage diversity in their microbiomes, regardless of their genetic relationships (7). Cohabitation has thus been reported as one of the main factors facilitating the asymptomatic transmission typical of SARS-CoV-2 (8), accounting for 45% of 793 new cases in Portugal in the period from 13 to 21 March 2020, according to the governmental Health National Service (9). Other forms of social contact were also relevant, namely, in companies (19%) and in nursing homes (11%). It seems reasonable to consider that in the context of group or family confinement, each individual may represent, in epidemiological terms, the entire group, as the individuals may share with each other many microorganisms of the community.

Although some recent studies suggest that life in the womb is not completely germfree (10–12), it has been argued that microorganisms do not colonize the baby (13, 14) and that the human microbiome begins to be enriched following birth and during the first 3 years of life. Vaginal birth and breastfeeding provide early contact with maternal microorganisms and help in the establishment of both gut and airway microbiomes, despite not being the main source of microbial diversity in adulthood (15–17). Skin-to-skin or mucous contacts happening while kissing and hugging are thus important sources of inoculation with human microorganisms from early life (18).

Also, contact with surfaces contaminated by humans can provide another important indirect source of colonizing microorganisms from one human microbiome to another. Babies and toddlers use their tongues to explore the household environment and, as a result, can ingest a wide variety of new microorganisms, some of which will potentially enrich their microbiota (19). The systematic disinfection of surfaces and hands can disrupt this indirect source of human microbial inoculation.

## SOCIAL DISTANCING AND THE HUMAN MICROBIOME

Interrupting the transmission of SARS-CoV-2 between individuals in a social network through confinement and adherence to rules of hygiene and social distancing has been important to contain COVID-19 spread, yet it also decreases the likelihood of sharing other microorganisms of the human microbiota. This decreases the repertoire of functional genes in our “other genome,” the gut microbiome (20), and could entail a loss of functions, exposing humans to disease (Fig. 1).

Many factors have been driving to a loss of bacterial diversity from one generation to the next in industrialized countries. Hygienic measures, vaccination, antibiotic use (21), and cesarean sections, among other factors, are contributing to a loss of our ancestral microbial heritage (15). Here, we postulate that the lack of contact between humans resulting from the social distance measures recommended for COVID-19 might also aggravate this situation and may increase the susceptibility of humans to disease.

## THE CLOSED LOOP BETWEEN DYSBIOSIS AND COVID-19

Already in 1969, Johanson and colleagues observed differences in the oropharyngeal bacterial microbiota in individuals with severe pneumonia, but those changes were not correlated with antibiotic administration or inhalation therapy, or with the duration of hospitalization (22). If, on one hand, critical illnesses and intensive care induce changes in the human microbiome, on the other, the changes in the lung and intestine microbiome also modulate critical diseases, as demonstrated in animal models and clinical trials (23). During lung infection, the induced shift in the microbiomes leads to a positive-feedback loop of inflammation and dysbiosis (23).

The healthy microbiome is closely related to the functioning of the immune system, and changes in the health status of the human host can have drastic effects on the microbiome, and vice versa (24, 25). Dysbiosis in the gut has been associated with many diseases, such as immune diseases like Crohn’s disease, ulcerative colitis, type 1 diabetes, celiac disease, allergy, and multiple sclerosis, metabolic diseases like obesity or type 2 diabetes and colorectal cancer, and autism (26). This is of particular relevance in elderly people that have a less diverse gut microbiota (5, 27).

It has been suggested that there is possible cross talk between the lung and the gut microbiota that could influence the outcome of COVID-19 (5). For example, dysbiosis of the gut microbiome can also increase the susceptibility to influenza virus infection in the lungs (5, 28).

We are led to question whether the recommended social distancing measures to prevent SARS-CoV-2 transmission could increase the number of other serious instabilities. The breaking of the contagion pathways reduces the sharing of microorganisms between people, thus favoring dysbiosis, which, in turn, may increase the poor prognosis of the disease.

## PERSON-TO-PERSON MICROBIAL TRANSMISSION AND ANTIBIOTIC RESISTANCE

It has been demonstrated that there is a positive correlation between the diversity of antibiotic resistance genes and the diversity of bacterial virulence genes in human metagenomes (29). Recently, using computer simulations, we have shown evidence that in a social network, bacterial transmission from one person to another is the major factor that explains this positive correlation between the diversity of antibiotic resistance genes and the diversity of virulence genes. Therefore, simply because people contaminate themselves in these social networks, we end up with the paradoxical and unwanted situation in which humans with a higher diversity of virulence genes in their metagenomes are precisely those expected to have a high diversity of resistance genes (30).

However, in some cases, antibiotic resistance entails a metabolic burden; hence, after antibiotic treatment ends, there is a decline of resistance by gene loss and competition with sensitive strains (31). This effect was highlighted in a metagenomic study in which it was observed that that the diversity of genes encoding antibiotic resistance in human intestinal microbiomes increases with age until reaching a limited level (32).

In this context, we trust that there could be a potential beneficial effect of social confinement in decreasing the spread of antibiotic-resistant microorganisms during antimicrobial therapy. This hypothesis needs to be tested experimentally and, if confirmed, could support new recommendations for the use of antibiotics.

## DISCUSSION

In this opinion article, we hypothesize that the social distancing imposed for COVID-19 prevention might have an impact on the diversity of the human gut microbiome and therefore on human health. We also argue that these measures could have two opposite consequences for COVID-19: (i) the loss of biodiversity, if not effectively restored, could be perennial and persist from one generation to the next, potentially driving to disease and a poorer prognostic of COVID-19, in a perverse and negative effect; (ii) social isolation and imposed hygiene rules can also lead to a decrease in the transmission of microorganisms and their genes from one individual to another, which might result in the dissociation of the correlation between the diversity of bacterial antibiotic resistance genes and virulence genes (29, 30). This can be a shorter-term positive effect that may not persist over time.

Social distancing implemented after the SARS-CoV-2 pandemic outbreak is therefore a double-edged sword. It might have both negative and positive impacts on the genomic dynamics of the human microbiome, and it is worth studying the implications for public health.

The development of these lines of research could help to provide national health systems with a comprehensive analysis of the confinement and social detachment effect on individual and community health. It could also support decision-making and measures to combat the pandemic, namely, in the definition of security protocols to control COVID-19 without compromising human health. Nevertheless, it is important to balance pandemic control with both the perverse effect of decreasing microbial diversity and the beneficial effect of decreasing the spread of antibiotic-resistant pathogenic bacteria (Fig. 1).
